# Chloral Hydrate in Pertussis

**Published:** 1873-02-15

**Authors:** J. F. Stair

**Affiliations:** Monticello, Wis.


### CHLORAL HYDRATE IN PERTUSSIS.

BY J. F. STAIR, M.D., MONTICELLO, WIS.

In the November number of the *Medical News and Library*, I noticed a communication from Dr. Henry, of Burlington, Iowa, on the use of chloral in whooping-cough, in which he stated that he had used it for two years with uniform success, never having met a single case in which it was not of benefit. On November 26th, 1872, I was called to see two little patients, brother and sister, who were suffering from whooping-cough, they having been attacked about two weeks previously. The girl, only seven months old, was apparently almost exhausted by the severity of the paroxysms. The boy, four years old, was also suffering severely, but the paroxysms were not as frequent—coming on, however, three or four times during the night with marked severity. Here, I thought, was a capital opportunity to give the chloral a trial. I accordingly ordered the following:

℞—Chloral hydrate, ʒ iij.

Spirits lavendulæ comp., ʒ i.

Syr. simp., ʒ vii.

Water, ℥ ii.—Mix.

To the boy was given two-thirds of a teaspoonful every four hours, and thirty drops were ordered to be given the babe at the same time. On visiting my patients the next day, I found the remedy had acted *like a charm*. In the case of the babe, the paroxysms of coughing had not recurred more than half as often as before the exhibition of the chloral, and were much modified in severity. The boy had had but one paroxysm of coughing in twenty-four hours. Under the use of the chloral they continued to improve, and within ten days were in no further need of treatment.

I own that my opportunities for testing the value of chloral in pertussis have been limited to these two cases; and I am aware we ought not to accredit great value to a remedy until a considerable induction of facts may warrant such claim; yet the cases given were fully up to the type, and for the reason that they yielded so *readily* to the treatment described, I have thought it my duty to communicate their history.

The spirits of lavender was added to the above prescription for the effect of its color. It imparts a beautiful pinkish hue to the mixture.

---

Death from Bichloride of Methylene.—At the Middlesex Hospital, London, Oct. 9th, a death occurred from the use of bichloride of methylene. The patient, male, aged 48, with a deep abscess in the buttock, and about to undergo an operation, inhaled about two drachms of the anæsthetic and died in one minute. Sylvester's method of artificial respiration and galvanism were resorted to, but without avail.

